# Parathyroid hormone assay standardization in CKD-MBD: resolving heterogeneity for precision medicine

**DOI:** 10.3389/fendo.2025.1702206

**Published:** 2025-11-18

**Authors:** Xuanchang Bai, Kaiduo Xu, Zijia Ma, Haijian Zhao, Weiyan Zhou, Chuanbao Zhang

**Affiliations:** 1 National Center for Clinical Laboratories, Institute of Geriatric Medicine, Chinese Academy of Medical Sciences, Beijing Hospital/National Center of Gerontology, Beijing, China; 2 Chinese Academy of Medical Sciences and Peking Union Medical College, Beijing, China

**Keywords:** PTH, CKD-MBD, heterogeneity, standardization, LC-MS/MS

## Abstract

Parathyroid hormone (PTH) plays a crucial role in calcium homeostasis and bone metabolism. Accurate measurement of PTH is essential for diagnosing and managing various endocrine and osteological diseases, particularly in the context of chronic kidney disease-mineral and bone disorder (CKD-MBD). Current immunoassays—categorized into three generations—struggle with PTH’s molecular heterogeneity. Mass spectrometry (MS) offers structural specificity, with recent advances achieving satisfactory sensitivity for intact 1–84 PTH quantification and identifying clinically relevant fragments. This review synthesizes the technological limitations of PTH measurement methods, highlights the critical standardization challenges, and discusses evolving strategies, including MS, to pave the way for reliable PTH testing in CKD management.

## 1 Introduction

Chronic kidney disease (CKD) is a global health burden, with CKD-mineral and bone disorder (CKD-MBD) as a major complication, affecting nearly all dialysis patients (1, 2). CKD-MBD encompasses a spectrum of clinical conditions, including secondary hyperparathyroidism (SHPT), bone disease, and vascular calcification, which significantly impact patient morbidity and mortality (3, 4). Parathyroid dysfunctions in this context drive perturbations in calcium, phosphorus, and vitamin D homeostasis, leading to debilitating bone pain, increased fracture risk, and accelerated cardiovascular disease progression (4, 5). Precise measurement of parathyroid hormone (PTH) is therefore critical, as it guides therapeutic interventions such as vitamin D analogs, phosphate binders, and calcimimetics, directly influencing disease trajectory and quality of life (6).

However, the clinical utility of PTH testing is compromised by the inherent molecular heterogeneity of circulating PTH and a lack of standardization across commercial assays (7, 8). This leads to poor inter-method comparability, risking misdiagnosis and inappropriate treatment (9–11). For instance, overestimation of biologically active PTH may prompt unnecessary surgical parathyroidectomy, while underestimation could delay interventions for progressive SHPT, exacerbating bone and vascular damage.

The International Federation of Clinical Chemistry and Laboratory Medicine (IFCC) Committee for Bone Metabolism has been working towards standardizing PTH assays, which is crucial for improving the consistency of result interpretation and establishing accurate reference ranges (12), but technological constraints persist. MS offers structural specificity for intact 1–84 PTH and fragment discrimination, yet broader implementation requires addressing sensitivity and cost barriers (13). This narrative review aims to synthesize the evolving landscape of PTH assay standardization. To ensure a comprehensive and unbiased perspective, the literature was surveyed using PubMed, and utilized key terms including “PTH,” “standardization,” “CKD-MBD,” “MS,” and “immunoassay”, without restriction on publication date, to encompass both seminal historical studies and the most recent advancements. The focus is placed on critically appraising the technological limitations, standardization challenges, and the clinical implications of assay heterogeneity in CKD-MBD management.õ

## 2 Biological basis and clinical necessity of PTH testing

PTH is synthesized as a preprohormone in the parathyroid glands and undergoes intracellular processing to form the mature 84-amino acid peptide (PTH 1-84). The secretion of PTH is primarily regulated by extracellular calcium concentration through the calcium-sensing receptor (CaSR) on parathyroid cells (14). Low calcium levels stimulate PTH secretion, while high calcium levels inhibit it. This regulation occurs in real-time, allowing for rapid adjustments in PTH levels. In the bloodstream, PTH exists in multiple forms including PTH 1-84,the biologically active, intact hormone with a short half-life of 2–4 minutes and other truncated PTH fragments, which constitute the majority of circulating PTH and have a longer half-life of 1–2 hours (15–20). PTH molecules are cleared from circulation through hepatic metabolism and renal clearance. The liver plays a crucial role in the peripheral metabolism of PTH 1-84, generating C-terminal fragments and the kidneys are primarily responsible for the disposal of C-PTH fragments.

**Figure 1:**
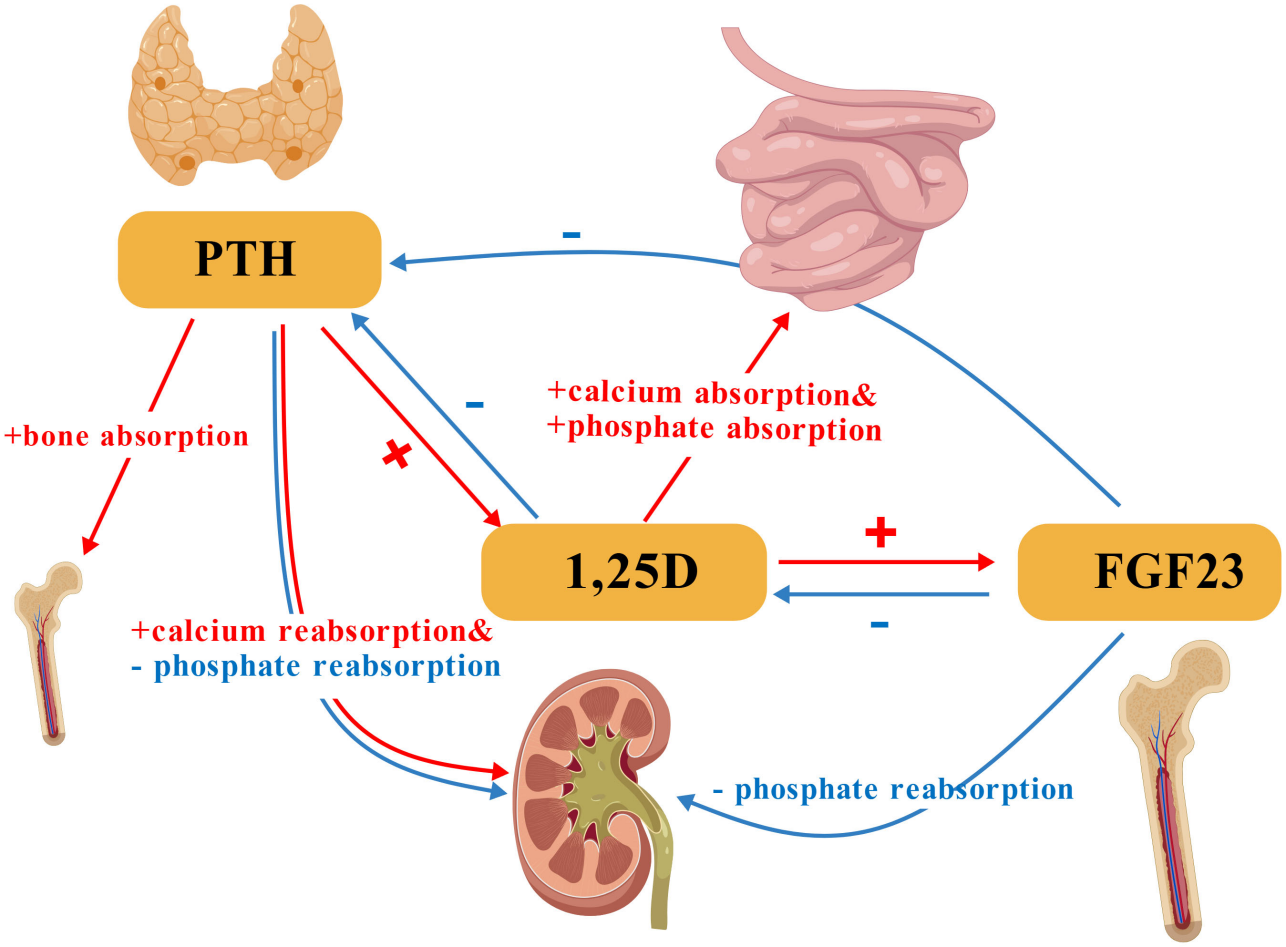
Core regulatory interactions of PTH, 1,25-dihydroxyvitamin D (1,25D), and FGF23 in calcium-phosphate homeostasis. PTH stimulates bone resorption and renal calcium reabsorption while promoting phosphaturia. 1,25D enhances intestinal calcium and phosphate absorption. FGF23 primarily promotes renal phosphate excretion. PTH, Parathyroid Hormone; 1,25D, 1,25-Dihydroxyvitamin D; FGF23, Fibroblast Growth Factor 23. Symbol and Arrow Definitions: “+”= Stimulation/promotion of the process;”-”: Inhibition/suppression of the process. Red arrows: Indicate stimulatory interactions. Blue arrows: Indicate inhibitory interactions.(Created with BioGDP.com).

PTH, in conjunction with vitamin D and fibroblast growth factor 23 (FGF23), primarily regulates calcium-phosphate homeostasis (**Figure 1**). This 84-amino acid polypeptide stimulates bone resorption to mobilize calcium, enhances renal calcium reabsorption while promoting phosphaturia, and activates vitamin D for intestinal calcium absorption (21–25).

Clinically, PTH measurement is essential for: (1) Diagnostic differentiation: Distinguishing primary hyperparathyroidism (PHPT, elevated PTH) from malignancy-associated hypercalcemia (22); (2) Disease stratification: Identifying SHPT in CKD where mineral dysregulation drives bone disease (26).; (3) Therapeutic monitoring: Guiding vitamin D/calcimimetic therapy in CKD and optimizing teriparatide dosing in osteoporosis (27–30); (4) Surgical confirmation: Validating successful parathyroidectomy through rapid intraoperative decline. However, the interpretation of PTH levels is complicated by physiological variations and the interplay with other factors. Establishing a single, universal reference interval is problematic. For instance, vitamin D status is a critical modifier: PTH levels begin to rise when 25-hydroxyvitamin D falls below a threshold, with estimates ranging from 15.8 ng/mL to 30 ng/mL, meaning that an elevated PTH may represent a physiological response to vitamin D insufficiency rather than a primary disorder (31, 32). Furthermore, renal function significantly modulates PTH, with a demonstrated stabilization point at an estimated glomerular filtration rate (eGFR) > 46.64 mL/min/1.73 m² (32). Recent studies have confirmed that PTH reference intervals must be stratified by age, gender, and body weight. Specifically, the upper reference limit is significantly higher in overweight subjects (31), and increases substantially with age, particularly in individuals over 70 years and in women compared to men of the same age group (32). These findings underscore that the application of a single reference range risks significant misclassification and highlights the necessity for context-dependent interpretation (33, 34).

## 3 Evolution of PTH detection method

**Figure 2:**
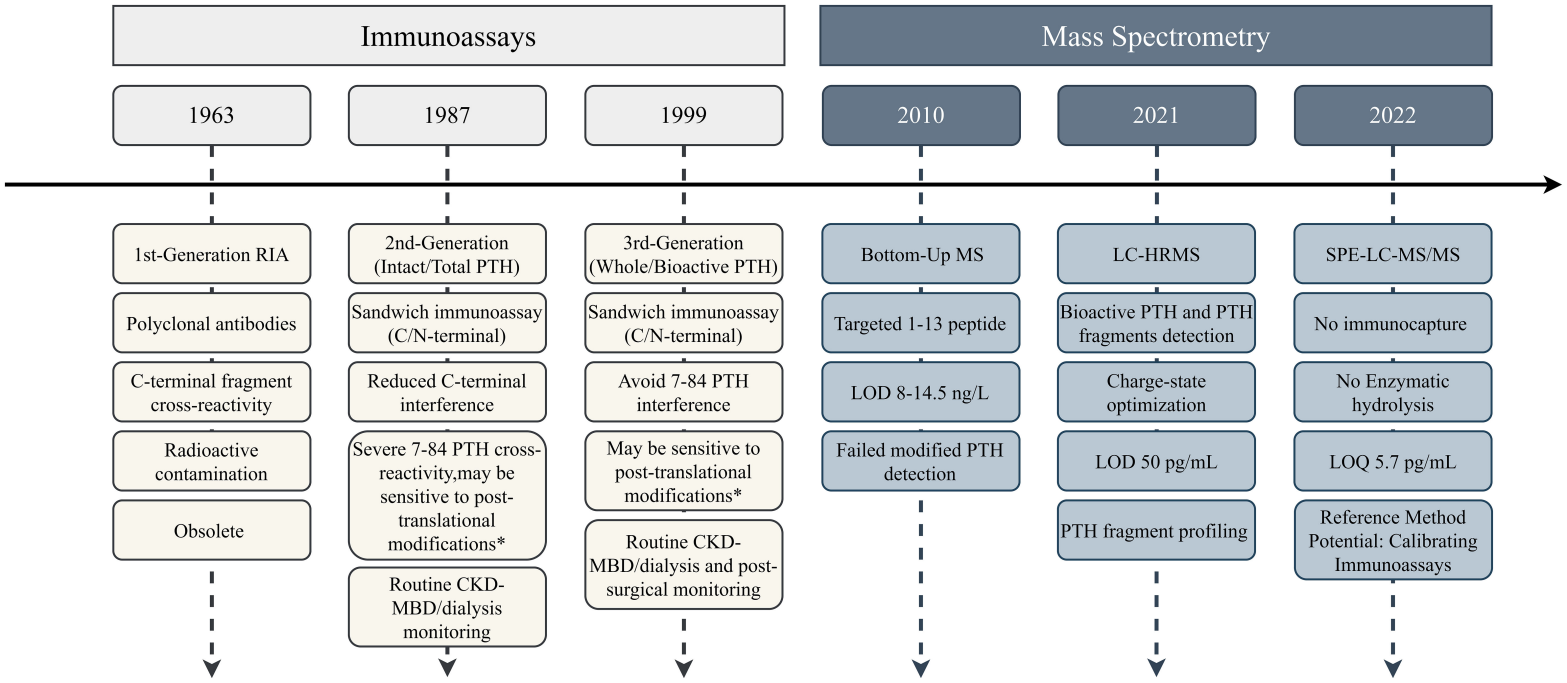
Timeline of PTH detection method evolution. Immunoassays progressed through three generations with incremental improvements in specificity, while mass spectrometry approaches focused on structural characterization capabilities. * Depends on the specific epitope. LOD, limit of detection; LOQ, limit of quantification; CV, coefficient of variation.

The evolution of PTH detection methods spans over five decades, with significant advancements in sensitivity, specificity, and clinical applicability (35). The chronological progression of PTH detection methodologies is summarized in **Figure 2**.

### 3.1 Evolution of immunoassays for PTH detection

PTH immunoassays have undergone three generations of technological refinement, with the primary goal of enhancing specificity for bioactive 1–84 PTH while minimizing cross-reactivity with inactive fragments (27).

#### 3.1.1 1st-generation immunoassays

The history of PTH measurement dates to 1963, when Berson et al. pioneered competitive radioimmunoassays (RIA) using polyclonal antibodies targeting mid-sequence epitopes (35, 36). These competitive assays measured immunoreactive PTH but lacked specificity for bioactive 1–84 PTH due to cross-reactivity with C-terminal fragments. Concurrently, assays utilizing antibodies against the N-terminal region were also developed (37, 38). These N-terminal assays provided valuable insights into PTH biology by targeting the biologically active part of the molecule, but their clinical utility was limited by the short half-life of N-terminal fragments in circulation and technological constraints of the era. Limitations of these early assays including radioactive hazards, prolonged incubation, and inability to distinguish fragments led to their obsolescence.

#### 3.1.2 2nd-generation immunoassays

The 1987 introduction of sandwich immunoradiometric assays (IRMA) marked a significant advancement. These employed two antibodies: a capture antibody targeting the C-terminal region (39–84 AA) and a ^125^I-labeled antibody binding to the N-terminal region (13–24 AA) (39). This design, along with later automated chemiluminescent and ELISA platforms, defined the “2nd-generation” of PTH immunoassays. These methods significantly reduced C-terminal fragment interference and demonstrated superior clinical correlation compared to 1st-generation RIAs, earning recognition as “intact/total PTH” assays in clinical guidelines.

Nevertheless, high-performance liquid chromatography (HPLC) studies revealed persistent cross-reactivity (up to 50%) with N-terminally truncated fragments in CKD patients (40, 41). Inter-assay variability from divergent antibody designs further compromised result comparability (42, 43).

#### 3.1.3 3rd-generation immunoassays

In 1999, Scantibodies Laboratory launched the first 3rd-generation IRMA kit, employing an N-terminal antibody targeting residues 1–4 to exclude 7–84 PTH interference (36). Termed “whole PTH” and “bioactive PTH” assays, these platforms retained C-terminal capture antibodies but introduced N-terminal antibodies specific to bioactive epitopes. Modern 3rd-generation assays have refined epitope design but face challenges from post-translationally modified PTH variants. These include phosphorylated amino-PTH (Ser17 phosphorylation) in parathyroid carcinoma and oxidized ox-PTH (Met8/18 oxidation) in dialysis patients under oxidative stress—both of which lose bioactivity yet cross-react with 3rd-generation and some 2nd-generation assays. This limitation has spurred efforts to develop fourth-generation assays insensitive to modified PTH forms (44, 45).

By analyzing China’s annual EQA data, PTH detection in the Chinese market is currently still dominated by 2nd-generation immunoassays from different manufacturers, with 3rd-generation immunoassays being less common. (**Table 1**).

**Table 1:** Major manufacturers of PTH testing on the Chinese market.

Method	Measurement principle	Capture antibody	Detection antibody	Analytical measurement range(pg/ml)	Reference Range (pg/ml)	LOD/LOQ (pg/ml)	Inter-assay CV	Traceability
Roche	ECLIA	6-32, Mouse Monoclonal Antibody	37-42, Mouse Monoclonal Antibody	1.2-5000	15-65	LOD=6	≤6.5%	Internal Commercial standard, and the recovery rate was evaluated using the NIBSC 95/646
Beckman	CMIA	1-34, Goat polyclonal antibody	53-68, Mouse monoclonal antibody	1-3500	12-88	LOD<4	≤5.6%	WHO 79/500 International Standard
Abbott	CMIA	1-34, Goat polyclonal antibody	39-84, Goat polyclonal antibody	3.0-3000.0	15-68.3	LOD<4	≤8.7%	WHO 79/500 International Standard
Siemens	CMIA	Mouse monoclonal antibody	Mouse monoclonal antibody	6.3-2000	plasma:18.4-80.1 serum:18.5-88.0	LOB=1.4 LOD=1.5	≤7.8%	Internal Commercial standard
Mindray	CMIA	\	\	\	12-88	LOD=3	≤10%	Internal Commercial standard
Snibe	CMIA	1-34, Mouse monoclonal antibody	50-70, Mouse monoclonal antibody	\	15-65	LOB ≤ 1 LOD ≤ 3	≤10%	NIBSC 95/646
Autobio	CMIA	\	\	\	11-81	LOB ≤ 2	≤15%	Internal Commercial standard
YHLO	CMIA	\	\	5-3000	15-65	\	≤15%	WHO 79/500 International Standard
Maccura	CMIA	1-34; Goat polyclonal antibody	39-84, Goat polyclonal antibody	5-5000	8-40	LOB ≤ 2.5 LOD ≤ 5	≤15%	NIBSC 95/646

ECLIA, electrochemiluminescence assay; CMIA, chemiluminescent microparticle immunoassay; CV, coefficient of variation; LOD, limit of detection; LOQ, limit of quantification.

### 3.2 Development of MS techniques for PTH detection

MS techniques have emerged as powerful tools for PTH detection, offering advantages in specificity and the ability to distinguish between various PTH fragments.

#### 3.2.1 Quantitative analysis of intact 1–84 PTH

Several studies have focused on developing liquid chromatography-tandem mass spectrometry (LC-MS/MS) methods for the quantification of intact 1–84 PTH. In 2009 Kumar et al. (46) introduced immunocapture-MS methods targeting the N-terminal 1–13 peptide (SVSEIQLMHNLGK) as a surrogate for 1–84 PTH with a limit of quantification (LOQ) at 39.1 pg/mL. Then Kritmetapak et al. (47) (2021) pioneered a dual-epitope immunocapture LC-HRMS method that directly measures 1–84 PTH without enzymatic digestion, achieving a linear range of 39.1–4,560 pg/mL with a LOQ at 50 pg/mL through DMSO-enhanced ionization. This breakthrough was followed by Farré-Segura et al.’s (2022) SPE-LC-MS/MS platform (48), which established unprecedented analytical performance with a 5.7 pg/mL LOQ and precision below 5.4% CV and met core reference method criteria. Most recently, Cao et al. (2025) (49)reported an LC-MS/MS method coupled with immunocapture for quantifying PTH 1–84 in patients with CKD. Li et al. (2025) (50)developed two complementary ID-MS approaches—amino acid IDMS (AA-IDMS) and peptide IDMS (peptide-IDMS) for accurate purity quantification of high-purity PTH materials. By targeting specific amino acids (Leu, Val, Phe) and a signature peptide (ADVNVLTK), both methods yielded consistent results, offering a promising framework for quantifying bioactive peptides with similar properties in clinical and research settings.

#### 3.2.2 Comprehensive fragment identification

The types and amounts of PTH fragments present in the human body have long been a focus of interest among researchers. Complementing these advances in the quantification of intact 1–84 PTH, MS has revealed the complex landscape of PTH fragments. Zhang et al. (51) (2006) first identified CKD-specific C-terminal fragments (34-84, 37-84, 38-84, 45-84) using capillary LC-MALDI-TOF/MS, while Lopez et al. (52) (2010) subsequently discovered novel truncations (28-84, 48-84, 34-77, 37-77, 38-77) through SRM-based immunoassays, quantifying key variants at detection limits of 8–22 pg/mL. In 2021, Kritmetapak et al. (47), established a high-resolution MS method for measuring PTH and its fragments. This approach allows for the simultaneous measurement of 1–84 PTH and various PTH fragments, providing a more comprehensive view of PTH metabolism, and has identified types of PTH fragments similar to those in the study by Lopez et al. **Table 2** summarizes the advances in PTH-related MS methods.

**Table 2:** Key research progress in MS methods for PTH.

Research team	Method	Research subjects	Immunocapture	Enzymatic hydrolysis	LOD/LOQ of 1–84 PTH(pg/mL)	PTH7-84	ox-PTH	Identified fragments/quantification
Zhang et al,2006 (51)	MALDI-TOF-MS or nano-LC-ESI-TOF-MS	Four patients with chronic renal insufficiency and six healthy women receiving recombinant human PTH	Goat polyclonal antibody against the C-terminal (39-84) region	NO	\	Not reported	Not reported	Four fragments were identified: 34-84, 37-84, 38–84 and 45–84 PTH.
Kumar et al,2010 (46)	LC-MS/MS	Primarily patients with hyperparathyroidism	Mouse monoclonal antibody against the C-terminal (44-84) region	YES	LOD=14.5;LOQ=39.1	Not reported (Adding up to 5μg/L of 7–84 PTH does not interfere with the quantification of 1–84 PTH by this method)	Not reported	Characterize 1–84 PTH by means of the 1–13 PTH peptide segment.
Lopez et al,2010 (52)	MALDI-TOF MS or SRM-MSIA	Twelve patients with severe renal impairment or end-stage renal disease (ESRD) and twelve healthy individuals	Goat polyclonal antibody against the C-terminal (39-84) region	YES	LOD=8; LOQ = 16	Not reported (The 7–13 peptide segment for characterizing 7–84 PTH was not found)	Not reported	Nine fragments were identified: 28-84, 34-84, 37-84, 38-84, 45-84, 48-84, 34-77, 37–77 and 38–77 PTH.
Kittrawee Kritmetapak et al,2021 (47)	LC-HRMS	221 patients with gradually declining renal function	Mouse monoclonal antibody against the C-terminal (44-84) region, monoclonal antibody targeting the N-terminal (26-32) region	NO	LOD =50	Not detected (LOD = 30pg/ml)	Not detected (LOD = 50pg/ml)	Eight fragments were identified: 28-84, 34-77, 34-84, 37-77, 37-84, 38-77, 38–84 and 45–84 PTH. Quantify PTH by using the full- length 1–84 PTH.
Jordi Farré-Segura et al,2022 (48)	LC-MS/MS	43 non-CKD patients, 28 CKD patients and 33 patients undergoing hemodialysis	Not used	NO	LOD=5.7	Not detected, but the separation of 7–84 PTH and 1–84 PTH was confirmed.	Not detected. The separation of ox-PTH and 1–84 PTH was confirmed.	Quantify PTH by using the full- length 1–84 PTH.
Cao et al,2025 (49)	LC-MS/MS	268 serum samples (non-CKD, CKD, hemodialysis patients)	NO	NO	LOD=5.0 pg/mL	Not reported	Not reported	Quantify PTH by using the full- length 1–84 PTH.
Li et al,2025 (50)	LC-MS/MS	High-purity PTH materials	Polystyrene beads coated with anti-PTH39–84 antibody	YES	\	Not reported	Not reported	Characterize 1–84 PTH by means of the 73–80 PTH peptide segment and Leu, Val, Phe

**Figure 3:**
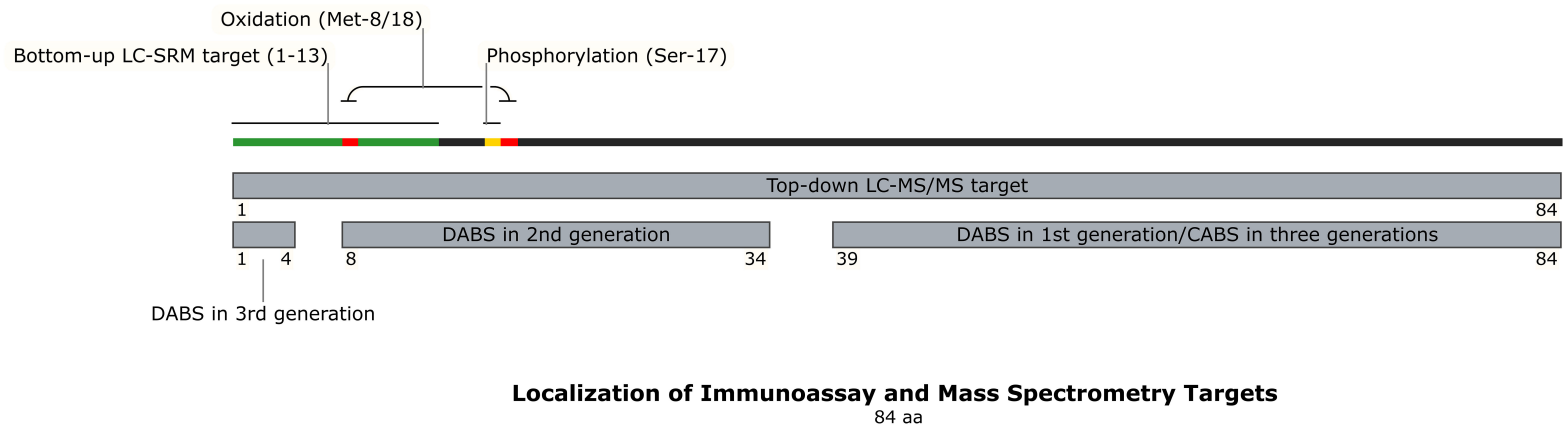
Localization of Immunoassay and Mass Spectrometry Targets. This schematic maps the binding regions of detection and capture antibodies used in different generations of PTH immunoassays (1st, 2nd, and 3rd gen) onto the 84-amino acid PTH structure. It also illustrates the target regions for bottom-up and top-down mass spectrometry analysis, and highlights key sites of post-translational modification (oxidation at Met8/18, phosphorylation at Ser17). The distinct molecular targets of each method explain why different assays yield non-comparable PTH results, especially in CKD where PTH fragment composition is altered. DABS, detection antibody binding site; CABS, capture antibody binding site; AA, amino acid.

Collectively, this methodological evolution demonstrates MS’s dual capability: establishing standardized quantification of bioactive 1–84 PTH while mapping clinically relevant fragment profiles. Nevertheless, challenges persist in detecting oxidized isoforms, streamlining workflows for clinical adoption, and reducing operational costs - critical frontiers for transforming MS from a reference technology to routine diagnostic tool. The localization of immunoassay and MS targets were described in **Figure 3** and **Table 3**.

**Table 3:** Comparative analysis of PTH detection methods.

Method	Target epitopes	Detected forms	Key limitations
1st-gen Immunoassay	Mid-region (44-68)/N-terminal region	1-84 +C-terminal fragments/N-terminal fragments	Low specificity for 1-84
2nd-gen Immunoassay	C39-84 + N13-24	1-84 + 7–84 fragments	Overestimates bioactive PTH
3rd-gen Immunoassay	C39-84 + N1-4	1-84 (excludes 7-84)	Cross-reacts with ox-/amino-PTH
MS(AA-MS)	Leu, Val, Phe	Leu, Val, Phe	Requires harsh conditions, is time-consuming and loses structural modifications
MS (Bottom-up)	1-13, 7-13, 73–80 peptides	Surrogate peptides	Loses structural modifications
MS (Top-down)	Intact 1-84	1-84 + modified forms	Requires ultra-high sensitivity

## 4 Interference factors in PTH detection and analysis

### 4.1 Heterogeneity of PTH and its impact on assays

**Figure 4:**
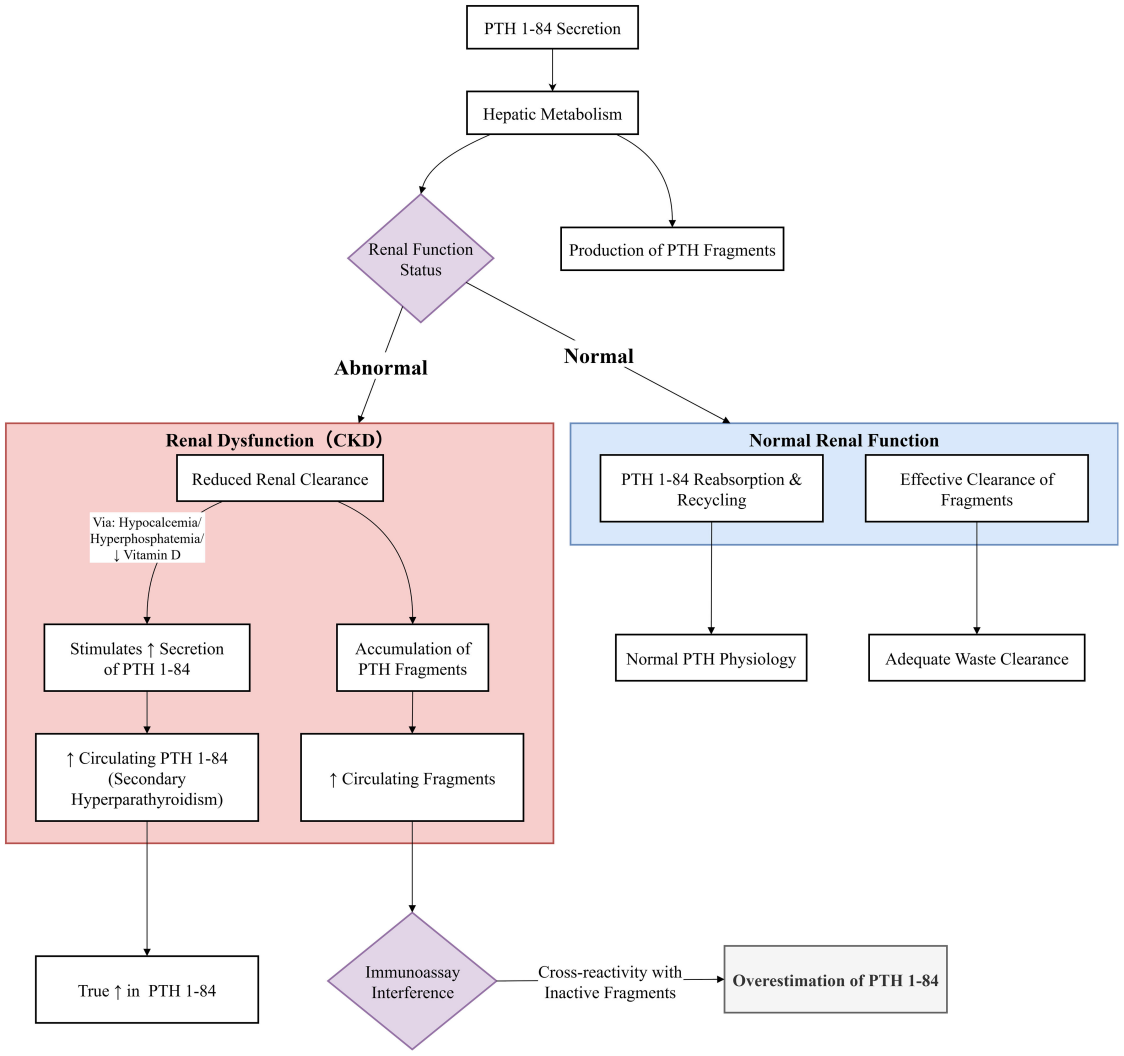
Renal clearance and assay interference of PTH fragments in chronic kidney disease. In normal kidney function, PTH fragments are efficiently cleared. In CKD, impaired renal function leads to the accumulation of these inert fragments. Commercially available immunoassays, particularly 2nd-generation ones, cross-react with these accumulating fragments. This results in a systematic overestimation of ‘intact PTH’ levels compared to the true concentration of bioactive 1–84 PTH (as measured by LC-MS/MS). This interference can mislead clinical decision-making, potentially resulting in the misclassification of bone turnover status and inappropriate therapy.

PTH heterogeneity refers to the coexistence of multiple molecular variants with structural and/or length differences in a single sample. These variants arise from alternative splicing, post-translational modifications, enzymatic processing, or degradation (21). The metabolic fate and clinical detection of these variants are largely influenced by renal function, as illustrated in **Figure 4**. As previously discussed, the discovery of PTH fragments and modified forms has driven immunoassay evolution. However, the full spectrum of circulating PTH variants remains incompletely characterized, with significant gaps in understanding their clinical relevance.

#### 4.1.1 Non-1–84 PTH: detection paradox and evolving clinical significance

Early studies using HPLC paired with immunoassays of varying specificities demonstrated the presence of non-1–84 PTH fragments in serum (53), suggesting that peptides starting at position 7 (i.e., PTH(7-84)) constituted a major component (40). The proportion of these immunoreactive fragments was consistently shown to increase with declining renal function (54). The development of 3rd-generation assays, which are insensitive to PTH(7-84), allowed for the estimation of a 1–84 PTH/7–84 PTH ratio based on the difference between 2nd- and 3rd-generation measurements. Initial studies proposed this inferred ratio as a potential predictor for adynamic bone disease in CKD patients (55).

However, this immunoassay-based picture and the clinical utility of the derived ratio are challenged by subsequent evidence. First, biological studies based on this model suggested that PTH(7-84) might antagonize the action of 1–84 PTH, leading to hypotheses about its role in skeletal resistance in CKD (54, 56, 57). However, recent sensitive MS methods have failed to consistently detect the canonical PTH(7-84) fragment in clinical samples (47, 48). This paradox, that inferred presence by immunoassay versus non-detection by MS, highlights a fundamental limitation in our current understanding of PTH heterogeneity. It suggests that the “non-1–84 PTH” measured by immunoassays may represent a heterogeneous mixture of uncharacterized fragments or that other interferences are at play.

Second, and consequently, the clinical value of the 1–84 PTH/7–84 PTH ratio has not been sustained. Subsequent research directly comparing this ratio with bone biopsy histomorphometry found no correlation with bone turnover categories, effectively refuting its initial promise for non-invasive diagnosis (58, 59). The fundamental uncertainty surrounding the exact molecules being measured, coupled with the lack of diagnostic power in validation studies, has led to the current consensus that measuring 1–84 PTH alone or its inferred ratio to non-1–84 PTH has limited value for the non-invasive diagnosis of renal osteodystrophy.

#### 4.1.2 Oxidized PTH: methodological challenges and biological questions

Methionine residues at positions 8 and 18 in PTH are susceptible to oxidation *in vitro*, particularly under conditions mimicking the elevated oxidative stress in dialysis-dependent CKD. Some 2nd-and 3rd-generation immunoassays, due to epitope designs, fail to distinguish ox-PTH from native non-oxidized PTH (n-ox-PTH), potentially leading to overestimation of bioactive PTH.

However, similar to the case with PTH(7-84), the actual presence and abundance of ox-PTH *in vivo* remains an open question. Although specialized methods like MS can theoretically enable specific detection, most clinical MS studies report an inability to detect ox-PTH or levels below quantification limits in clinical samples (47, 48). This disconnect may reflect rapid clearance, regulation by endogenous antioxidant systems, rather than being attributable to ex vivo oxidation during sample handling, which, in fact, would artifactually increase measured ox-PTH levels (60).

Consequently, while some early studies using specific immunological techniques associated ox-PTH with cardiovascular morbidity in CKD patients, its status as a direct pathogenic agent or a robust circulating biomarker is seriously questioned in the absence of robust MS validation (61–64). Current evidence suggests that the clinical utility of specifically quantifying n-ox-PTH is limited, as it has not been shown to be superior to conventional “intact PTH” measurements in predicting bone metabolism (65, 66).

#### 4.1.3 Amino-PTH: detection challenges and diagnostic value

In 2005, D’Amour et al. (53) combined HPLC with three immunoassays (targeting N-terminal 1-4, 15-20, and C-terminal 65–84 regions) to analyze serum from healthy individuals, PHPT, and CKD patients. They identified a novel PTH variant with an intact N-terminal (1-4) but modified 15–20 region, detectable by 3rd-generation “bioactive PTH” (CA-PTH) assays but not 2nd-generation “total PTH” (T-PTH) methods.

This variant, elevated in CKD, may contribute to the discrepancy between 2nd- and 3rd-generation assay results. While its role as a biomarker in CKD-MBD management remains unclear, its most significant clinical utility has been demonstrated in the differential diagnosis of parathyroid carcinoma, where an inverted ratio of 3rd-generation to 2nd-generation PTH (>1) shows high specificity (67–69).

### 4.2 Other interference factors

Immunoassays are the most widely used methods for PTH detection in clinical practice. However, they are subject to several interference factors. The most widely reported interference arises from endogenous heterophilic antibodies (70, 71). These antibodies, characterized by weak affinity and poly-specificity toward undefined antigens, bind nonspecifically to animal-derived assay components, generating false-positive or false-negative results. Heterophilic antibodies in patients may originate from exposure to animal products or vaccines containing animal serum/tissue derivatives. Additional interferences include biotin interference (72, 73),autoantibodies (74), anti-alkaline phosphatase antibodies (75) and PTH aggregation (76, 77). When aberrant PTH results are observed, interference can be investigated via linear dilution, chemical pretreatment or PEG precipitation.

## 5 Clinical translation of PTH detection methodologies

### 5.1 Intraoperative PTH monitoring

Intraoperative PTH (IOPTH) monitoring is a critical tool for confirming the success of parathyroidectomy. Its clinical utility hinges on two paramount factors: a rapid turnaround time (TAT) to guide real-time surgical decisions, and high analytical accuracy to correctly interpret PTH decay kinetics. The development of automated, rapid immunoassays has successfully addressed the speed requirement (78–80). These platforms enable surgeons to reliably observe the significant PTH drop following suspected adenoma resection within the operative timeframe. Further innovations, such as immunochromatographic test strips, aim to push detection speeds even further (81).

However, in patients with CKD, the accumulation of non-(1-84) PTH fragments with longer half-lives complicates IOPTH monitoring. 2nd-generation assays, which cross-react with these fragments, may yield a slower and less pronounced postoperative PTH decline, potentially leading to misinterpretation of incomplete resection or unnecessary exploration. In contrast,3rd-generation assays, designed to be specific for PTH 1-84, offer a distinct advantage in the CKD population. By excluding interference from inert fragments, these assays exhibit a more rapid and definitive PTH drop following successful adenoma resection, enhancing the predictive accuracy for surgical cure (82–84). This generational difference is minimal in patients with normal renal function, where fragment accumulation is negligible (84). Therefore, the clinical utility of third-generation assays in IOPTH is most pronounced in the context of CKD, directly impacting surgical decision-making and outcomes.

### 5.2 Variability and challenges of PTH assays in CKD-MBD management

The management of CKD-MBD is critically dependent on accurate PTH measurement, yet profound inter-assay variability undermines this foundation, leading to diagnostic inconsistency and therapeutic misalignment (63, 85, 86).

In CKD patients, 2nd-generation immunoassays systematically overestimate bioactive PTH by 30%-50% compared to 3rd-generation methods (84, 87, 88). A 2021 IFCC C-BM meta-analysis of 23 studies reported wide variability in method correlations (slopes: 0.50-2.2), attributed to fragment heterogeneity, evolving calibration standards, and operational differences. Notably, 3rd-generation assays exhibit improved inter-method agreement (slopes: 0.97-1.01) in CKD cohorts, though their clinical superiority remains debated (89, 90). Comparative studies between LC-MS/MS and immunoassays underscore the complexity of PTH detection. LC-MS/MS, which specifically targets the intact 1–84 PTH molecule, consistently reports lower PTH concentrations than immunoassays, yet the results from both methodologies show significant correlations (3, 4) (**Table 4**). The overestimation by immunoassays may be due to cross-reactivity with a broader spectrum of uncharacterized PTH variants present in CKD patients, and/or the compounding effect of classic immunoassay interferences, such as heterophilic antibodies, biotin, or pre-analytical sample handling artifacts.

**Table 4:** Comparison between MS and immunoassay.

Population	Y	X	Slope (a) [CI 95%]	Intercept (b) [CI 95%]	Reference
Pooled residual EDTA plasma samples	LC-MS-MS	Roche 2nd-generation	0.45	0.58	(47)
		DiaSorin 2nd-generation	0.21	7.21	
		Roche 3rd-generation	0.60	-7.1	
		DiaSorin 3rd-generation	0.73	-1.8	
		Fujirebio 3rd-generation	0.83	2.6	
43non-CKD, 48CKD, and 33DIA	LC-MS-MS	Fujirebio 3rd-generation	1.14 (1.09 to 1.22	−2.35 (−4.08 to −1.07)	(4)
EQCs (n = 35).		Fujirebio 3rd-generation	1.11 (1.05 to 1.18).	−0.15 (−1.80 to 1.95)	
43non-CKD, 48CKD, and 33DIA		Liaison 3rd-generation	1.33 (1.28 to 1.40)	−2.90 (−4.49 to −1.58	
EQCs (n = 35).		Liaison 3rd-generation	1.15 (1.08 to 1.22)	0.78 (−0.51 to 2.21)	
CKD3-5	LC-HRMS	Roche 2nd-generation	0.3375	29.315	(48)
CKD3		Roche 2nd-generation	0.551	28.111	
CKD4		Roche 2nd-generation	0.1742	44.261	
CKD5		Roche 2nd-generation	0.3634	11.172	

External quality assessment data further highlight inter-method variability in PTH detection. The 2015 UK National External Quality Assessment Service (NEQAS) PTH program revealed significant inter-method variability even for purified 1–84 PTH, highlighting calibration inconsistencies. Similarly, data from the 2024 National Center for Clinical Laboratories (NCCL) EQA survey indicated persistent inter-method discrepancies, even when using pooled blood samples from non-CKD populations as assigned value specimens—suggesting suboptimal comparability across PTH assay platforms.

As a result, substantial method-related variability in PTH results and the lack of clarity regarding recognized PTH metabolites create significant uncertainty. This discrepancy hinders the development of evidence-based clinical recommendations and critically impedes clinicians’ ability to determine if patients meet established guideline targets. The resulting ambiguity risks patient harm, including overtreatment or undertreatment (91, 92). Most crucially, it highlights the urgency and necessity of establishing the universally accepted common reference intervals and standardized treatment decision levels, which are fundamental for consistent patient management and therapeutic efficacy across healthcare settings.

### 5.3 Guideline evolution and regional disparities

Heterogeneity in CKD-MBD management guidelines reflects methodological limitations and regional epidemiological differences. The Kidney Disease Outcomes Quality Initiative(K/DOQI) framework (2003), based on 2nd-generation assays, recommended PTH targets of 150-300pg/mL to balance bone turnover risks (93). However, calibration drift with modern methods has rendered these thresholds obsolete. Kidney Disease: Improving Global Outcomes (KDIGO) guidelines (2009/2017), incorporating “U-shaped” mortality data, advocate broader targets (2-9× upper normal limits) to mitigate misclassification (94, 95). The Japanese Society for Dialysis Therapy (JSDT) guidelines, informed by survival analyses from its domestic dialysis registry, advocate stricter intact PTH targets (60-240pg/mL). This range correlates with reduced fracture and cardiovascular event rates in Japanese populations, possibly attributed to ethnic variations in the PTH-calcium response curve among Asian populations (96). In contrast, China’s Guidelines for Diagnosis and Management of CKD-MBD adopt the broader KDIGO-recommended range (2–9 times the upper normal limit), reflecting a paucity of high-quality randomized controlled trials (RCTs) in this domain. These guidelines emphasize context-specific adjustments based on regional healthcare resource availability. A comparative summary of international guidelines is provided in **Table 5**. Standardization remains a critical challenge. Current guidelines prioritize longitudinal trends over single measurements and advocate integrating biochemical markers for comprehensive assessment. Future efforts must focus on harmonized reference materials, method-specific decision thresholds, and multicenter RCTs to define optimal PTH targets across CKD stages. High-resource regions should accelerate adoption of 3rd-generation and MS-based assays to refine therapeutic precision.

**Table 5:** The target ranges of PTH in different guidelines.

CKD stages	K/DOQI	KDIGO	JDST	Chinese guideline for CKD-MBD
Stage 3	The optimal level of PTH remains unclear	The optimal level of PTH remains unclear	The iPTH range is 60-240pg/mL, and the wPTH range is 35-150pg/mL	The optimal level of PTH remains unclear
Stage 4				
Stage 5				
Stage 5D, patients receiving hemodialysis	150-300pg/mL	The iPTH level should be maintained at 2 to 9 times the upper limit of the normal value.	100-300pg/mL	The iPTH level should be maintained at 2 to 9 times the upper limit of the normal value.
Parathyroidectomy	\	In CKD stage 3-5D patients with severe hyperparathyroidism, if clinical/pharmacological treatments fail, parathyroidectomy is recommended.	It is recommended to perform parathyroidectomy on patients with severe SHPT (iPTH continuously > 500pg/mL; or wPTH > 300pg/mL.) who do not respond to medical treatment.	For patients in CKD stage 3-5D with severe SHPT (iPTH > 800pg/mL) that is unresponsive to medical treatment, parathyroidectomy is recommended.

iPTH, intact PTH(2^nd^-generation); wPTH, whole PTH(3^rd^-generation).

## 6 Standardization of PTH testing

### 6.1 Defining the measurand: navigating PTH heterogeneity

Circulating PTH exists as a heterogeneous mixture of molecular variants, with intact 1–84 PTH constituting only 20-30% of total PTH (64, 66, 97). The remainder comprises N-and C-terminal truncations and post-translationally modified forms. These fragments exhibit divergent metabolic pathways and biological activities. In CKD patients, impaired renal clearance leads to fragment accumulation, while oxidative stress in dialysis populations theoretically may promote oxidized PTH formation.

2nd-generation “intact PTH” assays, despite claims of specificity for 1–84 PTH, cross-react with 7–84 PTH, inflating reported values in CKD. 3rd-generation “whole PTH” assays mitigate this by targeting epitopes within residues 1-4. However, this N-terminal specificity does not protect against interference from other modified forms. Because the 1–4 epitope targeted by 3rd-generation assays remains structurally intact, these assays can still detect PTH molecules that have undergone downstream modifications such as oxidation (at Met8/Met18), leading to potential overestimation of bioactive hormone. Consensus is urgently needed to harmonize molecular specificity with clinical relevance. Current clinical priorities emphasize accurate quantification of intact 1–84 PTH as the primary measurand.

### 6.2 Reference measurement systems: establishing traceability

#### 6.2.1 Reference methods and reference materials

Currently, the Joint Committee for Traceability in Laboratory Medicine (JCTLM) database does not list any reference measurement procedures for PTH. Recent advancements in HRMS and multiple reaction monitoring (MRM) have significantly enhanced PTH assay standardization (47). Kritmetapak et al. utilized LC-HRMS to profile nine PTH fragments in CKD patients, achieving a detection limit of 50pg/mL. This method was designed with the capability to resolve oxidation variants through high-resolution accurate mass analysis, addressing cross-reactivity issues in immunoassays.

Farré-Segura et al.’s SPE-LC-MS/MS method with MRM achieves a lower limit of quantification (LLOQ) of 5.7pg/mL and precision (CV <5.4%). Despite unresolved challenges in detecting oxidized variants, its sensitivity and precision meet core criteria for an RMP, leading the team to propose it as a candidate reference method (48).

As for reference materials, the 1st International Reference Preparation (IRP 79/500), established in 1978 using human parathyroid tissue extracts, provided foundational standardization for PTH immunoassays. Its human-derived PTH fragments in an albumin/lactose matrix aimed to mimic physiological conditions, yet limitations persisted: indirect purity estimation, tissue-source fragment heterogeneity, and thermal instability. To address these challenges, the recombinant 1–84 PTH standard (NIBSC 95/646) was introduced as a successor, which serves as the primary calibrator for PTH assays. However, its limitations—including low purity [98.52 µg/vial by amino acid analysis (98)], matrix mismatch, and antibody-binding discrepancies—undermine commutability. Development of serum-based, unfrozen reference materials with demonstrated interchangeability is critical to bridging standardization gaps.

#### 6.2.2 Challenges in calibration

Inter-method variability persists despite traceability claims. Commercial calibrators, often synthetic PTH in animal serum matrices, exhibit divergent binding kinetics compared to human serum. A 2023 study comparing six major immunoassays demonstrated up to 40% variability in PTH values when calibrated against NIBSC 95/646, highlighting poor commutability (98). In China, most 2nd-generation assays trace to outdated standards (NIBSC 79/500) or internal commercial standards, while 3rd-generation assays (e.g., Roche) adopt NIBSC 95/646. Harmonization demands matrix-matched, human-derived reference materials validated by reference methods. Cavalier et al. (4) (2023) demonstrated the feasibility of standardizing PTH measurements through recalibration of immunoassays against a LC-MS/MS reference method calibrated with the WHO 95/646 International Standard. Their study recalibrated five PTH immunoassays (including second- and third-generation assays) using pooled plasma samples with LC-MS/MS-determined concentrations, resulting in a significant reduction in inter-assay variability.

### 6.3 Standardization efforts and IFCC working group initiatives

The IFCC established a Working Group for PTH, which later evolved into the Committee for Bone Metabolism (C-BM), to address the standardization of PTH assays (92, 99). The committee proposed a systematic approach to address the issues surrounding PTH measurement, including: a) Identifying the sources of variability in current PTH assays; b) Developing a reference measurement procedure; c) Establishing traceability to the International Standard; d) Harmonizing results across different assay platforms. To date, the working group has achieved some remarkable accomplishments including the establishment and calibration of an International Standard(NIBSC 95/646) and the development of a cRMP. To address global harmonization challenges, a collaborative roadmap is proposed, integrating regulatory bodies, healthcare facilities, and manufacturers. Regulatory bodies must enforce traceability by mandating IVD manufacturers to disclose calibration hierarchies, while EQA programs should transition to fresh-frozen serum proficiency panels to enhance methodological harmonization.

In China, NCCL has implemented a long-standing endocrine external quality assessment (EQA) program, encompassing PTH detection. In 2024, over 2,800 laboratories reported PTH results, almost utilizing 2nd-generation immunoassays. We distributed lyophilized human serum samples with varying concentrations to these laboratories across five levels. According to the 2024 EQA data, method-specific analysis demonstrated robust CVs ranging from 18.15% to 21.85%, with acridinium ester chemiluminescence, electrochemiluminescence (ECL), and AMPPD/luminol-based chemiluminescence dominating (80% of methods). As for manufacturers, Roche (30%), Abbott, and Beckman dominate the market, with 2nd-generation assays prevailing. The 2024 EQA data revealed inter-manufacturer CVs of 26.92-37.4% (excluding outliers beyond mean ±3 SD). These findings underscore persistent inconsistencies in PTH assay comparability across Chinese clinical laboratories. However, recent advancements in EQA programs have markedly improved harmonization. To address latent systemic errors, NCCL plans to launch a trueness verification program using commutable, value-assigned clinical samples.

Looking ahead, the ultimate goal of PTH standardization extends beyond analytical harmonization to ensure clinically meaningful interpretation. This necessitates the development of stratified reference intervals hat account for key physiological determinants. Robust evidence confirms that PTH reference values vary significantly with vitamin D status, renal function, age, gender, and body mass index. Future efforts must, therefore, focus on establishing well-defined, partitioned reference intervals for specific subpopulations to replace the current approach. Integrating these stratified intervals into clinical practice is paramount for transforming PTH from a mere number into a reliable tool for personalized patient management in CKD-MBD and beyond.

## 7 Conclusion

The clinical management of CKD-MBD is critically hampered by the lack of standardization in PTH measurement. The profound heterogeneity of circulating PTH fragments, combined with significant inter-assay variability, transforms a cornerstone biomarker into a source of diagnostic uncertainty and therapeutic risk. This review underscores the urgent need to bridge the gap between analytical sophistication and clinical utility.

To address these gaps, we propose a concerted focus on three critical fronts. First, technical standardization must accelerate: LC-MS/MS must be established and universally recognized as the reference method. Future development should focus on enhancing its sensitivity and resolving its capacity to detect post-translationally modified PTH variants. This will serve as the bedrock for creating commutable reference materials and defining a true, interference-free reference range for bioactive 1–84 PTH. Second, clinical guidelines must evolve from universal thresholds to method-specific, reference-range-aligned targets, anchored in IFCC-led harmonization of reference materials and traceability. This would transform vague recommendations into actionable, method- and stage-specific cutoffs, mitigating misclassification risks that currently drive overtreatment or undertreatment. Third, clinical practice should formalize integrated, longitudinal monitoring: combining PTH trends with calcium, phosphate, and bone-specific ALP, rather than relying on isolated measurements, to better reflect bone turnover and cardiovascular risk in CKD-MBD (100).

Ultimately, bridging analytical precision with clinical utility requires aligning technological advancement, reference range harmonization, regulatory framework, and clinical workflow. Only through such coordinated efforts can PTH testing transition from a source of uncertainty to a reliable cornerstone for personalized CKD-MBD management.
